# Rat cytochromes P450 oxidize 3-aminobenzanthrone, a human metabolite of the carcinogenic environmental pollutant 3-nitrobenzanthrone

**DOI:** 10.2478/v10102-010-0031-1

**Published:** 2010-11

**Authors:** Jana Mizerovská, Helena Dračínská, Volker M. Arlt, Jiří Hudeček, Petr Hodek, Heinz H Schmeiser, Eva Frei, Marie Stiborová

**Affiliations:** 1 Department of Biochemistry, Faculty of Science, Charles University, Prague, Albertov 2030, 128 40 Prague 2, CZECH REPUBLIC; 2 Section of Molecular Carcinogenesis, Institute of Cancer Research, Brookes Lawley Building, Sutton, Surrey SM2 5NG, UNITED KINGDOM; 3 Division of Molecular Toxicology, German Cancer Research Center, In Neuenheimer Feld 280, 69120 Heidelberg, GERMANY

**Keywords:** 3-aminobenzanthrone, cytochrome P450, HPLC, induction, inhibition, 3-nitrobenzanthrone, oxidation

## Abstract

3-Aminobenzanthrone (3-ABA) is a human metabolite of carcinogenic 3-nitrobenzanthrone (3-NBA), which occurs in diesel exhaust and air pollution. Understanding which cytochrome P450 (CYP) enzymes are involved in metabolic activation and/or detoxication of this toxicant is important in the assessment of an individual's susceptibility to this substance. The aim of this study was to evaluate the efficiency of rat hepatic CYPs to oxidize 3-ABA and to examine the metabolites formed during such an oxidation. The metabolites formed by CYPs in rat hepatic microsomes were separated by high performance liquid chromatography (HPLC). 3-ABA is oxidized by these enzymes to three metabolites, which were separated by HPLC as distinguish product peaks. Using co-chromatography with synthetic standards, two of them were identified to be oxidative metabolites of 3-ABA, *N*-hydroxy-3-ABA and 3-NBA. The structure of another 3-ABA metabolite remains to be characterized. To define the role of rat hepatic CYP enzymes in metabolism of 3-ABA, we investigated the modulation of its oxidation using different inducers of CYPs for treatment of rats to enrich the liver microsomes with individual CYPs. Based on these studies, we attribute most of 3-ABA oxidation in rat hepatic microsomes to CYP2B, followed by CYP1A, although a role of other hepatic CYPs cannot be ruled out. Inhibition of 3-ABA oxidation by selective inhibitors of individual CYPs, supported this finding.

## Introduction

**Figure 1:**
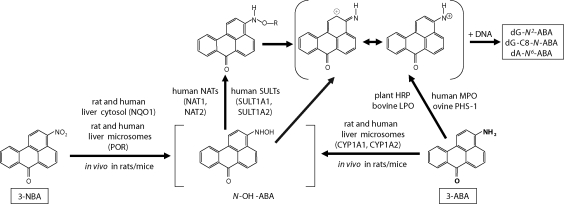
Pathways of metabolic activation and DNA adduct formation of 3-aminobenzanthrone and 3-nitrobenzanthrone. See the text for details. R=-COCH_3_ or -SO_3_H; dA-*N*^6^-ABA, 2-(2′-deoxyadenosin-*N*^6^-yl)-3-aminobenzanthrone; dG-*N*^2^-ABA, *N*-(2′-deoxyguanosin-*N*^2^-yl)-3-aminobenzanthrone; dG-C8-*N*-ABA, *N*-(2′-deoxyguanosin-8-yl)-3-aminobenzanthrone.

3-Aminobenzanthrone (3-ABA, Figure 1) is the reductive metabolite of the carcinogenic environmental pollutant, nitroketone 3-nitrobenzanthrone (3-nitro-*7H*-benz[*de*]anthracen-7-one, 3-NBA, Figure 1) (Hansen *et al*., 2007; Svobodová *et al*., 2007). In recent years 3-NBA has received much attention due to its presence in diesel exhaust and its extremely high mutagenic potency in the Ames Salmonella assay (Enya *et al*., 1997; Seidel *et al*., 2002; Arlt, 2005). 3-NBA is carcinogenic in rats, causing lung tumours after intratracheal instillation, and it is also a suspected human carcinogen (Seidel *et al*., 2002; Arlt, 2005; Nagy *et al*., 2005). The uptake of 3-NBA in humans has been demonstrated, because its metabolite 3-ABA has been found in urine samples of salt mine workers occupationally exposed to diesel emissions (Seidel *et al*., 2002). 3-ABA was also the main metabolite of 3-NBA formed in human fetal bronchial cells and rat lung alveolar type II cells (Borlak *et al*., 2000). In addition, 3-ABA was evaluated to be suitable for coloration of microporous polyethylene films, which are widely used for practical purposes such as separation of liquid mixtures, in particular, as separation membranes in chemical batteries (Grabchev *et al*., 2002), or an advantageous fluorescent phospholipid membrane label in the form of its *N*-palmitoyl derivative (Sykora *et al*., 2002). This suggests its industrial and/or laboratory utilization. Furthermore, genotoxicity of 3-NBA and 3-ABA has been documented by the detection of specific DNA adducts formed *in vitro* as well as *in vivo* in rodents in various tissues (Arlt *et al*., 2001; 2002; 2003a; b; c; 2004a; c; 2005; 2006b; Bieler *et al*., 1999; 2005; 2007; Stiborová *et al*., 2006; 2008). The predominant DNA adducts formed form 3-NBA and 3-ABA are *N*-(2′-deoxyguanosin-*N*^2^-yl)-3-aminobenzanthrone (dG-*N*^2^-ABA) and *N*-(2′-deoxyguanosin-8-yl)-3-aminobenzanthrone (dG-C8-*N*-ABA) and these are most probably responsible for the induction of GC to TA transversion mutations induced by these toxicants (Arlt *et al*., 2004a; Arlt *et al*., 2006b; Bieler *et al*., 2007).

Even though the epidemiological study on the toxicity of 3-ABA has not yet been evaluated, formation of DNA adducts by this reductive metabolite of 3-NBA *in vitro* and *in vivo* in rodents inducing these transversion mutations indicates its potential genotoxicity. Understanding which enzymes are involved in the metabolism (activation and/or detoxication) of 3-ABA is important in the assessment of susceptibility to this 3-NBA metabolite. Recently, we have found that cytochromes P450 (CYP) 1A1 and 1A2 are essential for 3-ABA oxidative activation in human and rat livers, forming the same DNA adducts that are formed *in vivo* by 3-ABA or 3-NBA (Arlt *et al*., 2004b). CYP1A1 is also efficient activator of 3-ABA in microsomes of rat kidneys and lungs, while prostaglandin H synthase (cyclooxygenase) plays a minor role in this subcellular fraction (Stiborová *et al*., 2008). Previous results also indicate that besides microsomal CYP enzymes cytosolic peroxidases might play a role in the oxidative activation of 3-ABA, mainly in extrahepatic tissues such as kidneys and lungs (Arlt *et al*., 2006a, Stiborová *et al*., 2006; 2008). In *in vitro* experiments, mammalian cyclooxygenase, lactoperoxidase and myeloperoxidase were found to be effective in activating 3-ABA (Arlt *et al*., 2006a) (Figure 1).

In contrast to the enzymes activating 3-ABA to species binding to DNA, those participating in 3-ABA oxidation to other potential metabolites have not been extensively studied so far. Therefore, here we investigated the oxidative metabolism of 3-ABA *in vitro*, in order to characterize the 3-ABA metabolites and to identify CYPs responsible for their formation. Hepatic microsomes of untreated (control) rats and those treated with two CYP inducers, namely, β-naphthoflavone (β-NF), which induces CYP1A and phenobarbital (PB), which induces CYP2B, were used for such a study. The selective inhibitors of individual CYP enzymes were also utilized to identify the most important enzymes oxidizing 3-ABA.

## Materials and methods

### Synthesis of 3-ABA and N-hydroxy-3-ABA (*N*-OH-ABA)

3-ABA and *N*-OH-ABA were synthesized as described (Arlt *et al*., 2003a) and their authenticity was confirmed by UV spectroscopy, electrospray mass spectra and high field proton NMR spectroscopy.

### Animal experiments and preparation of microsomes

The study was conducted in accordance with the Regulations for the Care and Use of Laboratory Animals (311/1997, Ministry of Agriculture, Czech Republic), which complies with Declaration of Helsinki. Microsomes from livers of ten male untreated Wistar rats and those of rats pretreated with β-NF (Sigma, UK) and PB were prepared by the procedure described previously (Stiborová *et al*., 2002). Rat liver microsomes contained 0.6 nmol CYP/mg protein. Hepatic microsomes of rats treated with β-NF and PB contained 1.3 and 1.5 nmol CYP/mg proteins, respectively.

### Incubations

Incubation mixtures, in a final volume of 500 µl, consisted of 100 mM potassium phosphate buffer (pH 7.4), 10 mM NADPH, 0.5 mg of microsomal protein and 5–50 µM 3-ABA (dissolved in dimethyl sulfoxide, DMSO). The reaction was initiated by adding 3-ABA. Incubations with rat microsomes were carried out at 37 °C for 5–180 minutes. Control incubations were carried out either (1) without the enzymatic system (microsomes) or (2) with microsomes and 3-ABA, but without NADPH. Then, 5 µl of 1 mM phenacetine in methanol was added as an internal standard and 3-ABA and its metabolites were extracted twice with ethyl acetate (2 × 1.5 ml). The extracts were evaporated to dryness; residues were dissolved in 30 µl of methanol and subjected to reverse-phase (RP)-HPLC to evaluate the amounts of residual 3-ABA and its metabolites.

### HPLC

The HPLC was performed with a reversed phase column (Nucleosil 100-5 C_18_, Macherey-Nagel, Duren, Germany, 25 cm×4.6 mm, 5 µm) proceeded by a C-18 guard column, using isocratic elution conditions of 70% methanol in distilled water with a flow rate of 0.6 ml/min. The HPLC was carried out with a Dionex HPLC pump P580 with UV/VIS UVD 170S/340S spectrophotometer detector set at 254 nm, and peaks were integrated with a CHROMELEON^TM^ 6.01 integrator.

### Inhibition studies

The following chemicals were used to inhibit 3-ABA oxidation in the presence of rat hepatic microsomes: α-naphthoflavone (α-NF), which inhibits CYP1A1 and 1A2, furafylline, which inhibits CYP1A2, diamantane, an inhibitor of CYP2B, sulfophenazole, which inhibits CYP2C and diethyldithiocarbamate, which inhibits CYP2E1 (Rendic and DiCarlo, 1997). Inhibitors were dissolved in 7.5 µl of methanol or water (in the case of diethyldithiocarbamate), to yield final concentrations of 0.01–0.1 mM in the incubation mixtures. Mixtures were then incubated at 37 °C for 5 min with NADPH prior to adding 3-ABA, and then incubated for a further 20 min at 37 °C. After the incubation, 3-ABA and its metabolites were extracted and analyzed by HPLC as described above.

## Results

**Figure 2:**
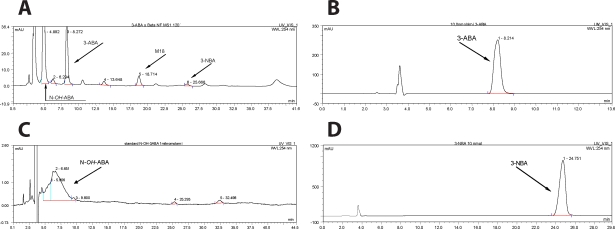
HPLC of 3-ABA metabolites produced by hepatic microsomes of rats treated with β-NF (A), HPLC of 3-ABA (B), *N*-hydroxy*-*3-ABA (C) and 3-NBA (D).

**Figure 3:**
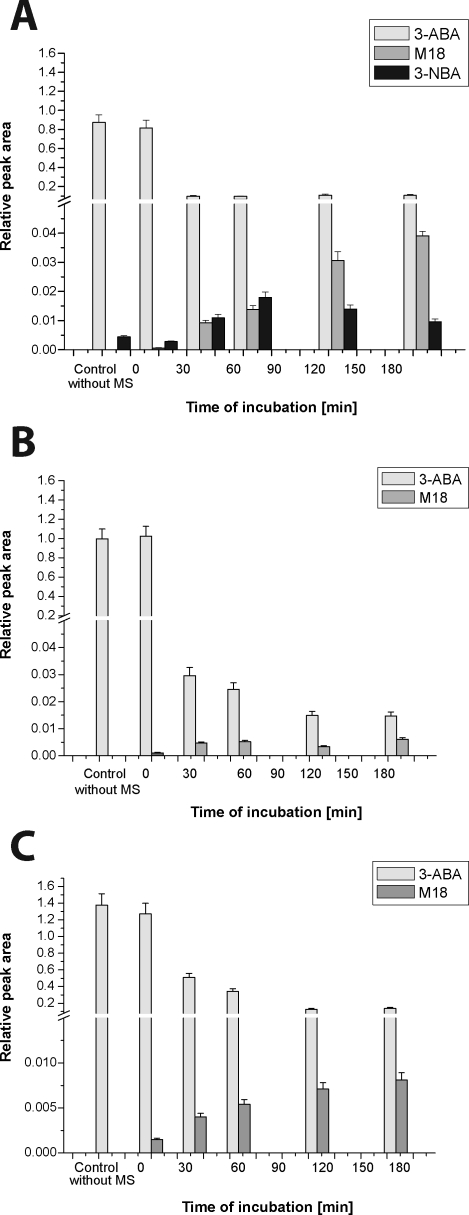
Time dependence of 3-ABA oxidation by hepatic microsomes of rats treated with β-NF (A), PB (B), and untreated (control microsomes) (C).

3-Aminobenzanthrone is oxidized by rat hepatic CYP enzymes in microsomes up to three metabolites (see Figure 2 for hepatic microsomes of rats treated with β-NF). These metabolites were separated by HPLC as distinguish product peaks (Figure 2A). Using co-chromatography with synthetic standards, two of them were identified to be the oxidative metabolites of 3-ABA (Figure 2B), *N*-hydroxy-3-ABA (Figure 2C) and 3-NBA (Figure 2D). Structures of another metabolite eluted with the retention time (r.t.) of 18 min, M18 (Figure 2A), remains to be characterized.

In order to evaluate the role of the rat hepatic CYPs in oxidation of 3-ABA, hepatic microsomes of rats treated with inducers of several CYPs (β-NF as an inducer of CYP1A1/2 and PB as an inducer of CYP2B) were used. All these microsomes as well as those of the untreated (control) rats were capable of oxidizing 3-ABA. Under the experimental conditions used, the most efficient microsomes oxidizing 3-ABA were those isolated from livers of rats treated with PB (rich in CYP2B), followed by those isolated from livers of rats treated with β-NF (rich in CYP1A) and by control microsomes (Figure 3). This finding suggests that CYPs of a 2B subfamily, followed by those of a 1A subfamily might play an important role in 3-ABA oxidation. Whereas hepatic microsomes rich in CYP1A (β-NF-microsomes) generated the final oxidative metabolite of 3-ABA, 3-NBA, this metabolite was not generated by other microsomes tested in this study. The metabolite with unknown structure, M18, was only formed from 3-ABA by these enzymatic systems (Figure 3)

The role of rat hepatic CYP enzymes in 3-ABA oxidation was further investigated by modulation of this reaction by selective inhibitors of individual CYPs. Of the inhibitors used in the study, diamantane, an inhibitor of CYP2B, was the most efficient inhibitor of 3-ABA oxidation with the IC_50_ value of 0.9 µM, followed by α-NF, sulfaphenazole and furafylline, inhibitors of CYP1A1/2, 2C and 1A2, respectively. The lowest inhibition efficacy was found for an inhibitor of CYP2E1, diethyldithiocarbamate. The IC_50_ values are shown in Table 1. These results supported the data found in the experiments with CYP inducers; CYP2B, followed by CYP1A, seem to be the most efficient in 3-ABA oxidation, whereas other CYP enzymes are less effective.

**Table 1:** The values of IC_50_ for inhibition of 3-ABA oxidation by CYPs.

Hepatic microsomes from rats pretreated with	Inhibitor	IC_50_ [μM]
β-naphtoflavone (CYP1A1/2)	α-naphtoflavone (CYP1A1/2)	10.9
β-naphtoflavone (CYP1A1/2)	Furafylline (CYP1A2)	16.6
Control	DDTC (CYP2E1)	96.8
Control	Sulfaphenazole (CYP2C)	10.8
Phenobarbital (CYP2B)	Diamantane (CYP2B)	0.9

## Discussion

3-ABA, the human metabolite of the ubiquitous environmental pollutant 3-NBA, was detected in the urine of smoking and nonsmoking salt mining workers occupationally exposed to diesel emissions at similar concentration (1–143 ng/24 h urine) to 1-aminopyrene (2–200 ng/24 h urine), the corresponding amine of the most abundant nitro-polycyclic aromatic hydrocarbons detected in diesel exhaust matter (Seidel *et al*., 2002). The present study has increased our knowledge on the potential of CYP enzymes to oxidize this toxicant and on the kinetics of such an oxidation.

The rat was used as an experimental model on the basis that the same enzymes activate 3-ABA in human and rat livers to species forming DNA adducts (Arlt *et al*., 2004b, 2006a; Stiborová *et al*., 2006). Therefore, the results should provide some indication of what might occur with 3-ABA in livers of humans. Rat and human hepatic CYP1A1 and/or 1A2 were found to be responsible for metabolic activation of 3-ABA (Arlt *et al*., 2004b). This finding corresponds to results found in the present study. Here, we have found that CYP1A enzymes are effective in oxidation of 3-ABA to the final oxidative metabolite of this compound, 3-NBA. Namely, to the metabolite that is formed through the formation of *N*-hydroxy-3-ABA. This reactive intermediate is, however, also decomposed to the ultimate carcinogenic species of 3-ABA, nitrenium and/or carbenium ions, forming 3-ABA-derived DNA adducts. Hence, this metabolic activation pathway seems to be the prevalent pathway of CYP1A enzymes. On the contrary, the CYP enzymes of the 2B subfamily (PB-microsomes), which were the most effective in oxidation of 3-ABA, did not form 3-NBA. Likewise, such a pathway was also not found for CYPs expressed in livers of untreated rats. Therefore, these CYP enzymes seem to play a detoxication role in 3-ABA metabolism. Nevertheless, this finding needs to be confirmed by additional studies. For example, the structural characterization of the 3-ABA metabolite M18 might shed some light on this feature.

In conclusion, the study showing for the first time identification of two metabolites formed from 3-ABA by CYP-mediated oxidation, confirmed the participation of CYP1A enzymes in activation of 3-ABA, namely, in formation of such a metabolite of 3-ABA, which is responsible for generation of 3-ABA-derived DNA adducts (N-hydroxy-3-ABA). Other CYPs expressed in rat livers are more important for detoxication of this compound. Structural characterization of the detoxication metabolite of 3-ABA awaits further investigation.
